# Initial Experience in Assessing the Stability of the Rist Guide Catheter for Transradial Neurointerventions

**DOI:** 10.7759/cureus.85432

**Published:** 2025-06-05

**Authors:** Yuki Kozaki, Kenji Fukuda, Syota Sakai, Kodai Matsuda, Fumiaki Fujihara, Tooru Inoue, Hiroshi Abe

**Affiliations:** 1 Department of Neurosurgery, Hakujyuji Hospital, Fukuoka, JPN; 2 Department of Neurology, Hakujyuji Hospital, Fukuoka, JPN; 3 Department of Neurosurgery, Fukuoka University Hospital, Fukuoka, JPN

**Keywords:** cerebral aneurysm surgery, chronic subdural hematoma (csdh), rist guide catheter, transradial approach, transradial neurointervention

## Abstract

Introduction: The Rist guide catheter is specifically designed for transradial neurointervention (TRN) and has demonstrated efficacy in accessing distal intracranial vessels, achieving a high success rate. We investigated the impact of catheter position on its stability during TRN.

Materials and methods: This retrospective study included 17 patients who underwent neuroendovascular procedures using the 7-French Rist guide catheter from March 2024 to February 2025. The procedures involved intracranial aneurysm and middle meningeal artery embolization. Catheter stability was evaluated based on its position and the effective catheter length (ECL), which is defined as the distance from the origin of the target vessel to the catheter tip.

Results: Stable catheter positioning was achieved in 13 out of 17 cases at the petrous segment of the internal carotid artery (ICA), the V3/4 segment of the vertebral artery (VA), and the distal external carotid artery (ECA) segment. The petrous segment corresponded to an ECL of approximately 20 cm, while the V3/4 or distal ECA segments corresponded to 16 cm. One case required switching to femoral access, but the procedure was successful in all cases, with no access site complications observed.

Conclusion: Our initial experience demonstrated that the position of the Rist catheter was relevant to its stability during TRN. Understanding its behavior would improve preprocedural planning and contribute to successful outcomes.

## Introduction

Neuroendovascular procedures for cerebrovascular diseases have been widely available as minimally invasive treatments [1]. Recently, transradial neurointervention (TRN) has been increasingly recognized for its potential advantages over traditional femoral access, such as fewer complications and faster recovery times [2,3]. The Rist guide catheter (Medtronic, Dublin, Ireland) is specifically designed for TRN and is a dedicated tool for these procedures [4]. Previous studies have demonstrated the Rist catheter's efficacy in accessing distal intracranial vessels, especially in challenging cases with tortuous vascular anatomy [5,6]. However, they have not provided a detailed description of the Rist catheter's position, particularly in relation to its stability. Investigating catheter positioning may contribute to preprocedural planning and facilitate intraoperative success by improving the predictability of catheter stability. The aim of this study was to investigate the influence of the position of the Rist catheter on stability during the procedure, with a particular focus on the depth of catheter placement.

## Materials and methods

Device description

The 7-French (F) Rist guide catheter (Medtronic, Dublin, Ireland) has an inner diameter of 0.079 inches and is available in lengths of 95 cm, 100 cm, and 105 cm. It features a flexible tip that gradually increases in stiffness from the tip to the proximal end over the first 25 cm. This design enables the catheter to smoothly navigate and reach intracranial vessels, rendering it suitable for complex neuroendovascular procedures. Additionally, according to the product specifications, the Rist catheter has two distinct transition zones, and it is regarded as being optimally stable when 14-17 cm or 19-21 cm from the tip is in the aortic arch.

Procedural technique

This retrospective study included 17 patients who underwent neuroendovascular procedures using the 7F Rist radial access guide catheter from March 2024 to February 2025. These procedures included eight intracranial aneurysm embolizations and nine middle meningeal artery embolizations (MMAE). All procedures were performed using a sheathless system. First, a 4F, 16-cm sheath was inserted into the radial artery. After confirming the suitability of the radial artery's course and diameter, the 0.035-inch, 180-cm guidewire was used to exchange the 4F sheath for the 7F Rist catheter. Regardless of the target vessel (right or left), the 105-cm Rist catheter was used in all cases to ensure sufficient working length, regardless of individual variation in body size or vascular anatomy. When radial artery spasm was observed, a 10-fold diluted isosorbide dinitrate solution was administered intra-arterially. Subsequently, the Simmons-type inner catheter was hooked at the origin of the target vessel. Then, the Rist catheter was advanced over a 0.035-inch guidewire. In cases requiring far distal advancement of the Rist catheter, especially those involving left-sided lesions, a 4.2 F FUBUKI catheter (Asahi Intec, Aichi, Japan) was used as a distal access catheter (DAC) in it to facilitate advancement and prevent arterial dissection. For cases involving flow diverter (FD) deployment, a DAC of a 5F Navien catheter (Medtronic, Minneapolis, MN) was used. Similarly, a DAC of 3.2F TACTICS catheter (Technocrat Corporation, Aichi, Japan) was used during the MMAE procedure.

Evaluation criteria

**Figure 1:**
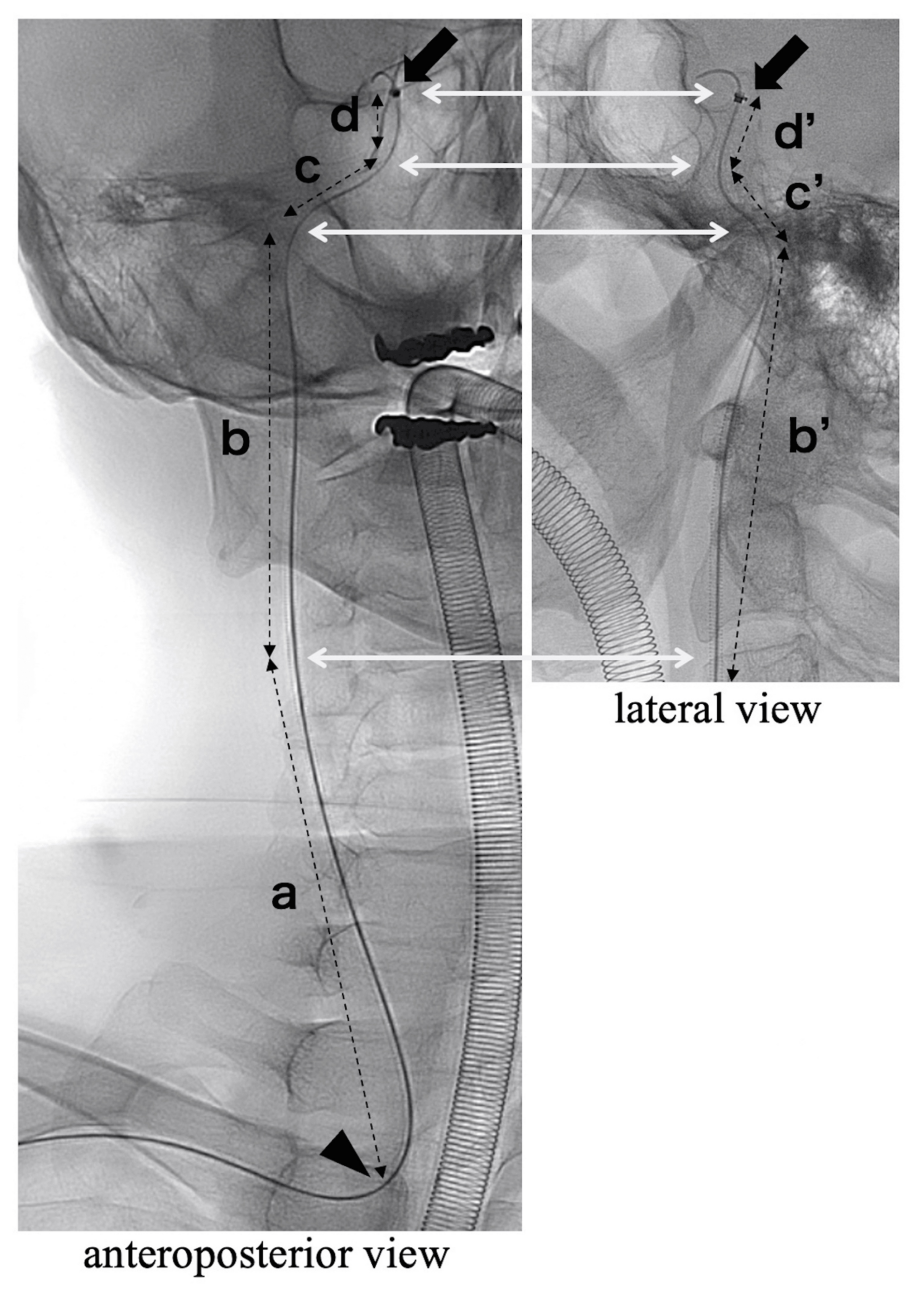
Method for estimating the effective catheter length Method for estimating the effective catheter length (ECL) using anteroposterior (AP) (left) and lateral (right) angiographic views. The catheter trajectory is divided by key turning points (a–d / b'–d') along the catheter’s path from the origin of the target vessel (arrowhead) to the catheter tip (arrow). Dashed lines connect these points to reflect the length of each segment of the catheter route. To estimate the total ECL, lengths between these points were measured in both AP and lateral views, and the longer value for each segment was adopted to compensate for vessel curvature and projection differences. In this case, the ECL was calculated as a + b′ + c + d′, selecting the longer measurement from either the AP or lateral view at each segment.

We evaluated the relationship between catheter instability and the position of the catheter tip during the procedures to assess the performance of the Rist catheter. Catheter instability was defined as downward slippage of the Rist catheter, such as falling into the aorta or subclavian artery, particularly after the insertion of the microcatheter, as judged by the operator. Additionally, to clinically validate the two transition zones described in the product specifications, we investigated the distance from the origin of the target vessel - the common carotid artery or the vertebral artery (VA) - to the catheter tip. We defined this length as the effective catheter length (ECL). The ECL was estimated based on preoperative anteroposterior (AP) and lateral angiographic views (Figure 1). The catheter trajectory was divided into segments according to visible turning points, where the catheter changed direction due to vessel curvature. Straight lines were drawn between these points, and the segment lengths were summed to calculate the ECL. When discrepancies existed between AP and lateral views, the longer total length was adopted as the estimated ECL to better reflect the actual path length through tortuous vessels. Due to the two-dimensional nature of the images and the anatomical curvature of the vessels, particularly in the thoracic region, the ECL values represent approximate linear distances and not true 3D path lengths. Therefore, ECL should be considered a reference index to compare catheter position relative to its transition zones, rather than an exact anatomical measurement. The type of aortic arch, access side, and use of DAC were analyzed for each procedure. Access site complications, procedure-related complications, and procedure success were also recorded. The study was approved by the ethics committee of our hospital.

## Results

The mean age of the patients was 74.8 years (range: 53-90 years), and most were male (10 males and seven females). The mean radial artery diameter on angiographic evaluation was 2.6 mm (range: 2.1-3.1 mm); the right artery was used in 16 cases, and the left artery was used in one case. There were seven cases of coiling, one case of FD placement for aneurysms, one case of MMAE for a transverse/sigmoid dural arteriovenous fistula, one case of MMAE for a brain tumor, and seven cases of MMAE for chronic subdural hematoma (CSDH). Table 1 summarizes all detailed procedures, including anatomical variations, the position of Rist catheter placement, and its stability.

**Table 1:** Summary of cases treated using the Rist catheter AcomA: anterior communicating artery, AN: aneurysm, BA: basilar artery, BAT: balloon assist technique, CCA: common carotid artery, CSDH: chronic subdural hematoma, DAC: distal access catheter, DAVF: dural arteriovenous fistula, DCT: double catheter technique, ECA: external carotid artery, ECL: effective catheter length, F: female, FD: flow diverter, ICA: internal carotid artery, L: left, M: male, MCA: middle cerebral artery, MMAE: middle meningeal artery embolization, Para: paraclinoid, Pcom: posterior communicating artery, R: right, SAT: stent assist technique, VA: vertebral artery

Case	Age	Sex	Diagnosis	Procedure	Access side	Type of aortic arch	Rist catheter placement	ECL	DAC	Stability
											1	61	M	R/MCA AN	Coiling (BAT)	R	2	ICA petrous	20cm	−	yes
2	82	F	AcomA AN	Coiling (BAT)	L	3	ICA petrous	21cm	−	yes
3	53	F	L/ICA Pcom AN	Coiling (BAT)	L	2	ICA petrous	20cm	−	yes
4	70	M	R/ICA para AN	Coiling (SAT)	R	1	ICA petrous	20cm	−	yes
5	70	F	L/ICA PcomA AN	Coiling (DCT)	L	3	ICA petrous	20cm	−	yes
6	54	F	R/ICA para AN	FD	R	1	ICA cervical	16cm	+	no
7	71	F	BA AN	Coiling (BAT)	R	3	VA V4	16cm	−	yes
8	50	M	R/VA AN	Coiling (SAT)	R	1	VA V3	15cm	−	yes
9	76	M	R/DAVF	MMAE	R	1	Distal ECA	16cm	+	yes
10	70	M	R/meningioma	MMAE	R	3	Distal ECA	15cm	+	yes
11	79	M	R/CSDH	MMAE	R	2	Distal ECA	17cm	+	yes
12	79	M	L/CSDH	MMAE	L	2	Distal ECA	17cm	+	yes
13	84	M	R/CSDH	MMAE	R	1	Distal ECA	17cm	+	yes
14	84	M	L/CSDH	MMAE	L	1	Distal ECA	17cm	+	yes
15	84	M	L/CSDH	MMAE	L	3	CCA	6cm	+	no
16	90	F	R/CSDH	MMAE	R	3	CCA	10cm	+	no
17	90	F	L/CSDH	MMAE	L	3	CCA	10cm	+	no

**Figure 2:**
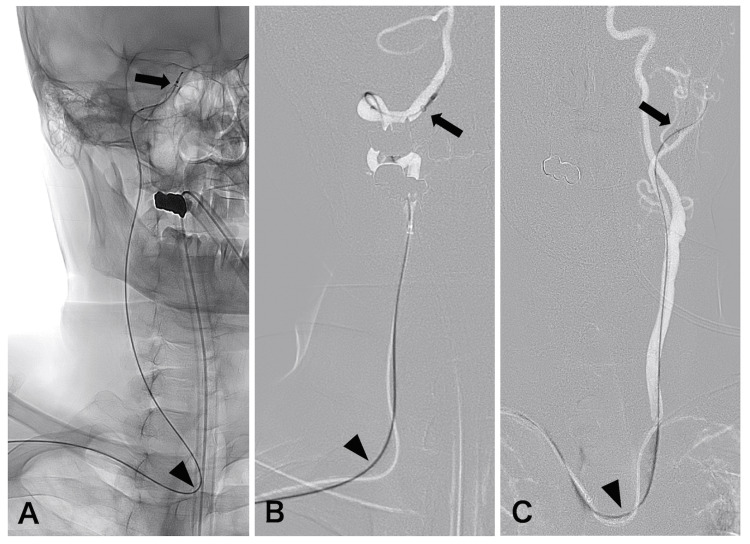
Cases with stable Rist catheter positioning (A) Angiogram showing the right internal carotid artery (ICA) paraclinoid segment aneurysm treated using a stent-assisted technique. The Rist catheter (arrow) was positioned at the petrous segment of the ICA, which corresponds to an effective catheter length (ECL) of approximately 20 cm from the origin of the common carotid artery (arrowhead). (B) Angiogram showing the basilar artery aneurysm treated using a balloon-assisted technique. The Rist catheter (arrow) was positioned at the V4 segment of the right vertebral artery, which corresponds to an ECL of approximately 16 cm from the origin of the vertebral artery (arrowhead). (C) Angiogram showing a middle meningeal artery embolization for a left chronic subdural hematoma. The Rist catheter (black arrow) was positioned at the distal segment of the external carotid artery, which corresponds to an ECL of approximately 17 cm from the origin of the common carotid artery (arrowhead).

**Figure 3:**
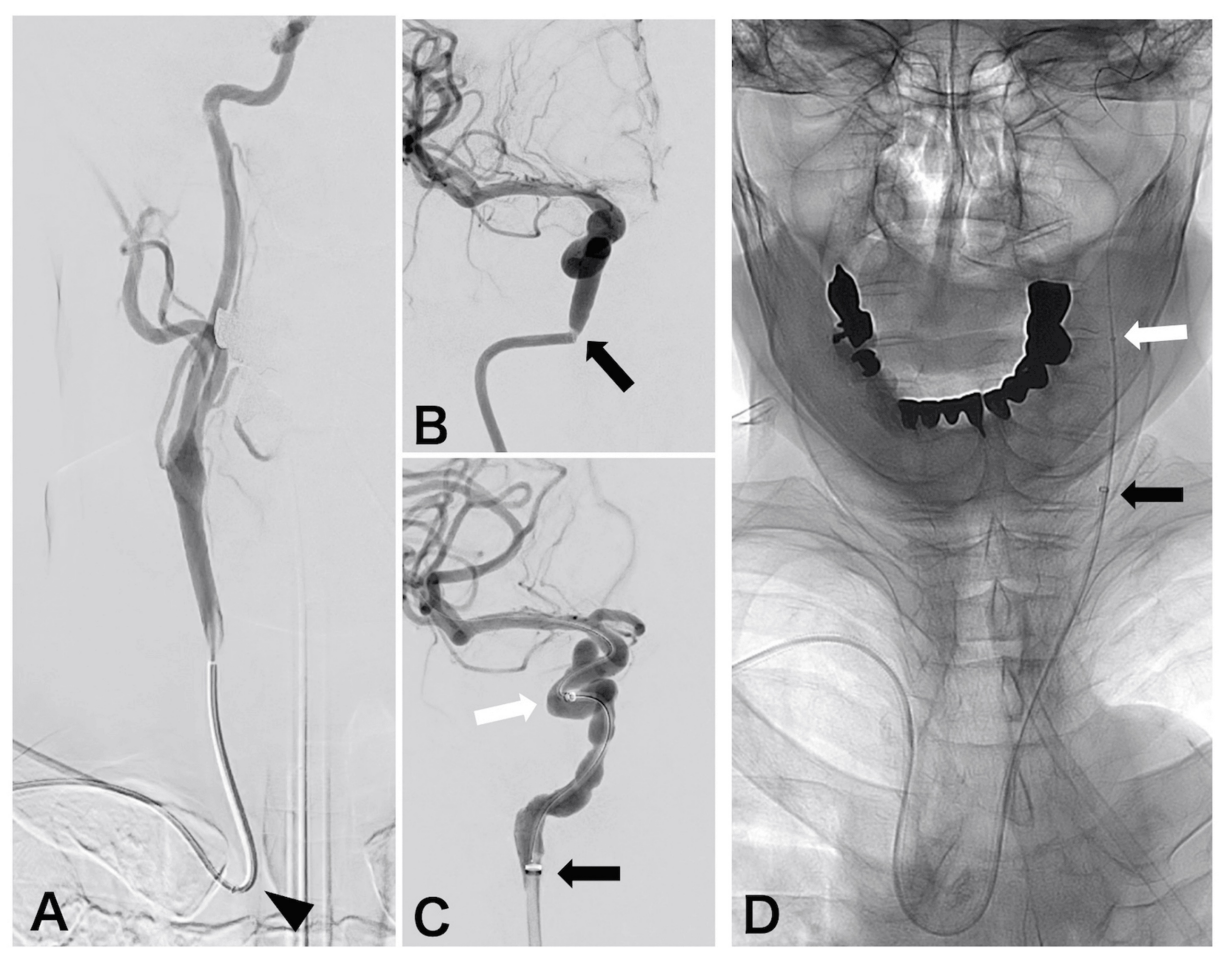
Cases with unstable Rist catheter positioning (A) Angiogram showing a right internal carotid artery (ICA) paraclinoid segment aneurysm, treated with a flow diverter. It reveals a relatively straight segment from the origin of the common carotid artery (black arrowhead) to the cervical ICA, lacking any significant vessel curvature. (B) A spasm was identified at the petrous segment of the ICA (black arrow). (C) The Rist catheter (black arrow) was retracted to the cervical segment (corresponding to an effective catheter length (ECL) of 16cm). The procedure was successfully performed due to the support of a 5F distal access catheter (DAC) (white arrow). (D) Angiogram showing a middle meningeal artery embolization for a left chronic subdural hematoma. The Rist catheter (black arrow) slid downwards to the common carotid artery, which corresponds to an ECL of 10 cm. The procedure was successfully performed due to the support of 3.2F DAC (white arrow).

The Rist catheter demonstrated stability during the procedures in 13 of the 17 cases. In internal carotid artery (ICA) access, the catheter exhibited stable performance when positioned at the petrous segment (Figure 2A). Even when navigated above the petrous segment, the 7F Rist catheter settled back to the petrous segment during the procedure. For posterior circulation access, stability was maintained at the V3-V4 segment of the VA (Figure 2B). For external carotid artery (ECA) access, stability was achieved at the distal segment of the ECA, which refers to the segment just proximal to the origin of the superficial temporal artery (Figure 2C). Specifically, the petrous segment corresponded to an ECL of approximately 20 cm, while the V3/4 segment and distal ECA segment corresponded to an ECL of approximately 16 cm. In these cases, the relationship between the access side, the type of aortic arch, and catheter stability was not clear. In one case of a right ICA aneurysm, spasm occurred at the petrous ICA segment, prompting the catheter to be lowered to the high cervical segment (Figures 3A-3C). The Rist catheter exhibited instability during FD placement; however, the use of a DAC enabled successful completion of the procedure. In this case, the ECL was 16 cm. In three cases of CSDH (two with left-sided lesions and one with a right-sided lesion), particularly in elderly patients with a type 3 aortic arch, catheter navigation was challenging. The catheter could only be positioned up to the carotid bifurcation, where the Rist catheter tended to slide down. Nevertheless, the procedures were successfully completed with the assistance of a DAC (Figure 3D). The ECL was 6 cm and 10 cm in these cases, respectively.

In one case of a left ICA aneurysm, the Rist catheter kinked at the aortic arch during coil embolization using the double catheter technique, necessitating a switch to transfemoral access. All procedures were completed successfully, and no complications related to the procedures or the access sites were observed. In all cases, the patency of the radial artery was confirmed using a portable ultrasound immediately after the procedure and at the three-month follow-up.

## Discussion

Recent catheter technology has provided the ability to ensure stability during procedures by varying the stiffness of the catheter. The introduction of the Rist guide catheter, specifically designed for TRN, has presented significant advantages in neuroendovascular procedures, especially for radial access [4-6]. Interestingly, the Rist catheter has specific transition zones at two key points, around 15 cm or 20 cm from the catheter tip, for optimal stability in the aortic arch, as indicated by the product specifications. This study is the first to demonstrate the relationship between the stability of the Rist catheter and its positioning in clinical practice.

In our cases, the catheter exhibited stable positioning at the petrous segment of the ICA during aneurysm treatment. The catheter was also stabilized at the V3-4 segment of the VA or at the distal ECA. The petrous segment corresponded to an ECL of about 20 cm, while the V3/4 and distal ECA segments corresponded to 16 cm. The findings of this study align with the product specifications concerning the transition zone. Once the Rist catheter is navigated to this position, stable catheter behavior can be expected. However, one case involving the ICA demonstrated instability despite an ECL of approximately 16 cm. Anatomical review revealed that she had a relatively straight segment from the aortic arch to the cervical ICA, lacking any significant vessel curvature. This is likely caused by insufficient mechanical support along the catheter trajectory, resulting in downward slippage of the Rist catheter. This observation suggests that the presence of one or more anatomical curvatures can enhance catheter stability by providing additional support against proximal migration [7]. Understanding these features would lead to improving preprocedural planning and ensuring successful treatment.

On the other hand, the procedure becomes unstable if the Rist catheter cannot be guided to the optimal position, as observed in our CSDH cases. The complexity of catheter navigation is associated with several factors, including the access side, the presence of vascular tortuosity, and the configuration of the aortic arch [8]. However, even when the catheter's behavior becomes unstable, the use of a DAC can improve stability and support the Rist catheter, allowing the procedure to be completed successfully. In fact, in the report by Rautio et al., DAC use was observed in 76 cases (76%), primarily for aneurysm treatments and MMAE [6]. When catheter navigation is expected to be difficult, it is important to consider using a catheter system that allows the use of DACs.

The Rist catheter is less suitable for carotid artery stenting (CAS) procedures, in which the guiding catheter is usually positioned in the common carotid artery. Furthermore, for thrombectomy in acute ischemic stroke, one drawback of using the Rist catheter is that it cannot be used with balloon guiding, and large-bore suction catheters cannot be employed. The transradial approach has been shown to be comparable to the transfemoral approach with regard to clinical outcomes when limited to the use of balloon-guided catheter [9-11]. Thus, the Rist catheter was rarely used for CAS and thrombectomy [5,6].

Although previous reports have utilized the 7F Rist catheter with a 7F sheath, we employed it in a sheathless manner. It has an outer diameter of 2.33 mm, which is comparable to using a 5F sheath. It is important for the immediate and long-term preservation of the radial artery to increase the radial-to-sheath ratio in TRN [12]. Recently, Allard et al. reported that six out of 22 patients (27.3%) presented with delayed radial artery occlusion after intracranial aneurysm treatment using the 7F Rist catheter with sheath [13]. While employing a sheathless guiding catheter may increase the risk of medial dissection, any radial artery injury remains comparable to that observed with conventional sheath usage [14]. Notably, no significant access-site complications were observed in this study, indicating that the sheathless approach may be especially helpful for the prevention of radial artery occlusion.

Although our study demonstrates the efficacy of the 7F Rist catheter, it is important to acknowledge its limitations. First, the study focuses solely on the 7F version of the Rist catheter. Due to the catheter’s design, the optimal ECL may differ with different sizes of the catheter. In particular, the 6F Rist catheter may provide sufficient stability in procedures that require a smaller profile, such as MMAE. However, for intracranial aneurysm, which requires more delicate techniques, the 7F catheter is more suitable, especially when using a DAC. Comparative studies with other radial-specific catheters could also provide deeper insights into the Rist catheter's relative strengths and limitations [15]. Second, we did not measure the height and weight of the patients. Therefore, there may be individual variations in the length of the target vessel and the catheter's position. However, our findings regarding stability at ECLs of 16 cm or 20 cm can help predict a stable location for each patient by measuring the distance from the target vessel’s origin using preoperative angiograms. Third, the estimation of ECL may have been affected by vessel tortuosity and anatomical overlap, as it was based on two-dimensional digital subtraction angiography (DSA) images. Although three-dimensional imaging modalities such as CTA would allow for more precise measurements, we did not perform them routinely due to concerns about additional contrast load and radiation exposure. Future studies incorporating 3D imaging may provide a more accurate assessment of the relationship between catheter length, positioning, and stability. Finally, this study is a single-center, retrospective study with a small sample size. Nevertheless, we believe that understanding the behavior of the Rist catheter, especially its positioning, could significantly improve preprocedural planning and contribute to successful outcomes.

## Conclusions

In our initial experience, we demonstrated that positioning the 7F Rist catheter based on the catheter tip location and ECL was important for maintaining stability during TRN. Stable positioning was observed at the petrous ICA with an ECL of approximately 20 cm and at the V3-V4 segment of the VA or distal ECA with an ECL of approximately 16 cm. Anticipating catheter behavior based on preprocedural imaging can help achieve optimal positioning and improve procedural efficiency. These practical insights are expected to facilitate the broader and safer use of radial-specific catheters in neuroendovascular procedures.
